# Circulating Endothelial Progenitor Cells and Clinical Outcome in Patients with Aortic Stenosis

**DOI:** 10.1371/journal.pone.0148766

**Published:** 2016-02-25

**Authors:** Sara Shimoni, Iris Bar, Valery Meledin, Estela Derazne, Gera Gandelman, Jacob George

**Affiliations:** 1 The Heart Institute, Kaplan Medical Center, Rehovot, Israel, Affiliated to the Hebrew University and Hadassah Medical School, Jerusalem, Israel; 2 Sackler Faculty of Medicine, Tel Aviv University, Tel Aviv, Israel; Centro Cardiologico Monzino, ITALY

## Abstract

**Background:**

Aortic stenosis (AS) is the most common valvular disease. Endothelial progenitor cells (EPCs) have a role in the repair of endothelial surfaces after injury. Reduced numbers of EPCs are associated with endothelial dysfunction and adverse clinical events, suggesting that endothelial injury in the absence of sufficient repair by circulating EPCs promotes the progression of vascular and possibly valvular disorders. The aim of this study was to assess EPC number in patients with AS and to study the predictive value of their circulating levels on prognosis.

**Methods:**

The number of EPCs was determined by flow cytometry in 241 patients with AS and a control group of 73 pts. Thirty-eight, 52 and 151 patients had mild, moderate and severe AS, respectively. We evaluated the association between baseline levels of EPCs and death from cardiovascular causes during follow up.

**Results:**

EPC level was significantly higher in patients with AS compared to the control group (p = 0.017). Two hundred and three patients with moderate and severe AS were followed for a median of 20 months. One hundred and twenty patients underwent an intervention. Thirty four patients died during follow up, 20 patients died due to cardiac causes. Advanced age, the presence of coronary artery disease, AS severity index (combination of high NYHA class, smaller aortic valve area and elevated pulmonary artery pressure) and a low EPC number were predictors of cardiac death in the univariate analysis. Multivariate logistic regression model identified low EPCs number and AS severity index as associated with cardiac death during follow up (p = 0.026 and p = 0.037, respectively).

**Conclusions:**

EPC number is increased in patients with AS. However, in patients with moderate or severe AS a relatively low number of EPCs is associated with cardiac death at follow up. These results may help to identify AS patients at increased cardiovascular risk.

## Introduction

Degenerative aortic valve (AV) stenosis (AS) is the most common valvular disease and increases in prevalence with age.[1] Severe aortic valve stenosis accounts for considerable morbidity and death, especially in older patients. Aortic valve stenosis is the primary indication for valve replacement in Western countries, and the number of interventions continues to increase as the population grows older. However, despite improved outcome due to valvular interventions, AS continues to be a prevalent disease with substantial morbidity and mortality and with no effective treatment strategy to inhibit progression of AS.

Bone marrow is the origin of subsets of circulating stem cell populations which can differentiate into the endothelial lineage. Numerous studies have shown that circulating progenitors are reduced in disorders associated with compromised endothelial function and atherosclerosis.[2,3] Lower number of circulating endothelial progenitor cells (EPCs) are related to endothelial dysfunction and adverse clinical events in patients with atherosclerosis, suggesting that endothelial injury in the absence of sufficient repair by circulating EPCs may promote the progression of vascular disease. [3,4]

AS which was attributed for years to a passive wear and tear process, is now recognized as an active inflammatory and potentially modifiable pathology, with similarities to atherosclerosis.[5–7] The surface of valve leaflets is covered with endothelial cells, which are critical in maintaining a non-thrombogenic surface and for the transduction of mechanical and biochemical signals.[8] Mature endothelial cells possess a limited regenerative capacity.[9] Thus, there is growing interest in EPCs, especially in their role in the maintenance of endothelial integrity and function.[10,11] During the development and progression of AS, the endothelial cell layer is damaged followed by infiltration of inflammatory cells, which can induce a vicious cycle leading to progression of the disease and valvular calcification. Loss of endothelial integrity, as well as calcification occurs primarily on the aortic side of the valve leaflet. [12–14]

EPCs are present in degenerative aortic valves and degenerative bioprosthesis, particularly in aorto-luminal regions of injured cusps, whereas non diseased valves are free of EPCs.[15] However, the role of circulating EPCs in AS is not well established. In addition, there is no data on the prognostic value of EPCs in patients with AS. Several clinical studies that included a low number of patients, showed contradictory results with regard to circulating EPCs in patients with significant AS.[16–18] The aim of this study was to assess circulating EPC numbers in a larger cohort of patients with AS and to study for the first time, the predictive value of circulating EPC levels on prognosis in these patients.

## Methods

### Patients

The study included 250 consecutive patients with AS who were followed in the valvular disease clinics in Kaplan Medical Center between July 2011 and December 2013. Patients were compared with 75 consecutive patients with similar atherosclerotic risk factor profile who had no significant valvular disease. Patients with more than mild to moderate aortic regurgitation were excluded. Patients with mitral stenosis and more than mild mitral regurgitation were excluded. Patients with a history of acute coronary syndrome or revascularization in the previous 3 months or any type of malignant or hematologic disorder were also excluded. This study was approved by the Kaplan Medical Center institutional ethics committee on 11/07/2011 and all patients provided written informed consent.

### Assessment of risk factors

Risk factors were assessed in all patients based on their medical records. Diabetes mellitus was defined as hyperglycemia requiring pharmacologic therapy; hypertension was diagnosed as either a systolic or a diastolic increase in blood pressure (>140/90 mm Hg) or use of antihypertensive therapy; hypercholesterolemia was defined as a total cholesterol level of greater than 200 mg/dL or use of lipid-lowering agents; and cigarette smoking as being an active smoker or having a smoking history of at least 10 pack-years. Coronary artery disease (CAD) was defined as a history of myocardial infarction or presence of CAD on coronary angiography.

### Follow up

Patients were followed in the valvular clinic. The indication for intervention was determined by patient symptoms and co-morbidities, based on standard guidelines.[19] The primary end point of the study was cardiac mortality. Causes of death were determined by examination of hospital records and medical files of patients' general practitioners. Deaths due to cardiovascular causes included sudden deaths and deaths from acute myocardial infarction, CAD or congestive heart failure (CHF).

### Echocardiography studies

Transthoracic echocardiography including assessment of the aortic valve was performed according to established guidelines.[20,21] Left ventricular volumes and left ventricular ejection fraction (LVEF) were assessed by modified Simpson's method. Left ventricular mass was assessed using the Devereux formula (0.8{1.04[([LVEDD + IVSd + PWd]^3^ − LVEDD^3^)]} + 0.6). Aortic valve area was calculated using the continuity equation.[22] Mild, moderate and severe AS was defined as valve area of 1.5–2.0 cm^2^, 1.0–1.5 cm^2^ and less than 1.0 cm^2^, respectively.

### Quantification of circulating endothelial progenitor cells by flow cytometry

Peripheral blood was drawn from patients with AS and control donors. Circulating EPCs were defined as CD34pos/KDRpos cells and quantified by flow cytometry. 7.5x10^6^ mononuclear cells were isolated from peripheral blood by density gradient centrifugation, and were incubated at 4°C in the dark for 30 min with mouse anti-human KDR-APC, mouse anti human CD34-PE, and their isotype controls (Miltenyi Biotec, Bergisch Gladbach, Germany) as previously described.[23] After incubation, cells were washed with phosphate buffered saline, and analyzed by FACS (LSRII, Becton Dickinson). For a clear analysis, at least 50,000 total events or 1000 CD34pos/KDRpos events were collected by flow cytometry. The number of EPCs was calculated as an absolute number of cells per μL blood.[24,25] Since the measuring of EPC levels is not standardized test, the patients were categorized into two groups according to the median endothelial progenitor-cell count at the time of enrollment.[4]

### Statistical analysis

Continuous data are presented as medians and interquartile ranges (25^th^-75^th^ percentiles) for skewed distributed variables or as mean ± SD when normally distributed. Categorical data are presented as absolute numbers with their respective percentages. Chi-square test was used for categorical variables and Student’s *t* test for continuous variables. Logistic regression analysis was used to assess the association between AS and baseline characteristic variables. One way Anova test was used for comparison of EPC level in patients with different AS severities and AS etiologies and in different NYHA class levels. End point analysis based on Cox proportional-hazards model was used to analyze the data. Since AVA, NYHA class and pulmonary artery systolic pressure are related clinically and statistically (r = -0.427, p<0.001, r = -0.28, p<0.001 and r = 0.402, p<0.001, respectively)) we performed factor analysis to calculate a variable that included the 3 variables and used it as the AS severity index. A multivariate proportional-hazards regression analysis was performed to determine the association between endothelial progenitor-cell counts and each outcome. Overall survival was calculated using Kaplan-Meier test and subgroups were compared with log rank test. Cardiac death was calculated by cumulative incidence analysis with death due to other causes considered as a competing risk. All the above analyses were considered significant at p≤0.05. Analyses were performed with IBM SPSS version 21 and R2.14.2 software packages.

## Results

### Patient characteristics

The initial study group included 325 patients. EPC levels could not be evaluated in 11 patients due to technical problems. The study population included 241 consecutive patients (age 77± 10years, 95 male) with AS who were followed in the valvular heart clinic. A control group included 73 stable patients (age 70± 12years, 47 male) with no valvular disease who were referred to coronary angiography for chest pain evaluation or patients with atrial arrhythmia referred to echocardiography. Thirty-eight, 52 and 151 patients had mild, moderate and severe AS, respectively. The etiology of AS was degenerative in 211 patients, bicuspid aortic valve in 24 patients and rheumatic in 6 patients. Patient baseline characteristics are outlined in Table 1. Patients with AS were older, more women and had higher frequency of treatment with beta blocker agents and furosamide. The hemoglobin level was lower and the LV mass was higher in patients with AS.

**Table 1:** Patient's baseline characteristics.

	No AS	AS	p
	N = 73	N = 241	
Age	70±12	77±10	0.001
Gender (male), n (%)	47 (64.38)	95 (39.42)	<0.001
Hypertension n (%)	48 (65.75)	194(80.50)	0.011
Diabetes mellitus n (%)	22 (30.14)	84 (34.85)	0.48
Hyperlipidemia n (%)	56 (76.71)	177 (73.44)	0.65
Smoker n (%)	6 (8.22)	34 (14.11)	0.23
Coronary artery disease n (%)	33 (45.21)	106 (43.98)	0.89
Aspirin n (%)	38 (52.05	117 (48.55)	0.7
Beta blockers n (%)	36 (49.32)	158 (65.56)	0.014
ACEI n (%)	31(42.47)	123 (51.04)	0.23
Statin n (%)	48 (65.75)	169 (70.12)	0.47
Furosamide n (%)	7 (9.59)	46 (19.09)	0.07
Hemoglobin (g/dL)	13.6±1.39	12.32±1.64	0.001
White blood cells (K/ul)	7.3±2.02	7.49±1.92	0.45
Platelets (K/ul)	217±80	218.01±77.25	0.9
Creatinine (mg/ dL)	0.96±0.26	1.00±.34	0.43
Total cholesterol (mg/ dL)	164.25±38	167.28±39.27	0.55
LDL cholesterol (mg/ dL)	92.94±31	94.58±31.75	0.72
Triglycerides (mg/ dL)	129.39±63.5	125.23±57.61	0.62
HDL cholesterol(mg/ dL)	50.19±15	50.87±14.51	0.76
Left ventricular end diastolic diameter (mm)	45.66±5.84	45.60±5.64	0.94
Left ventricular end systolic diameter (mm)	27.63±5.82	27.01±5.20	0.57
Left ventricular ejection fraction (%)	55.11±6.43	55.31±7.00	0.84
Left ventricular mass (gr)	182.78±48	222.88±60.48	<0.001
EPCs (cells/μl)	2.64 (1.59–4.94)	4.08 (1.39–7.6)	0.017

ACEI-angiotensin converting enzyme inhibitor

### Endothelial progenitor cells in patients with AS and in the control group

**Fig 1:**
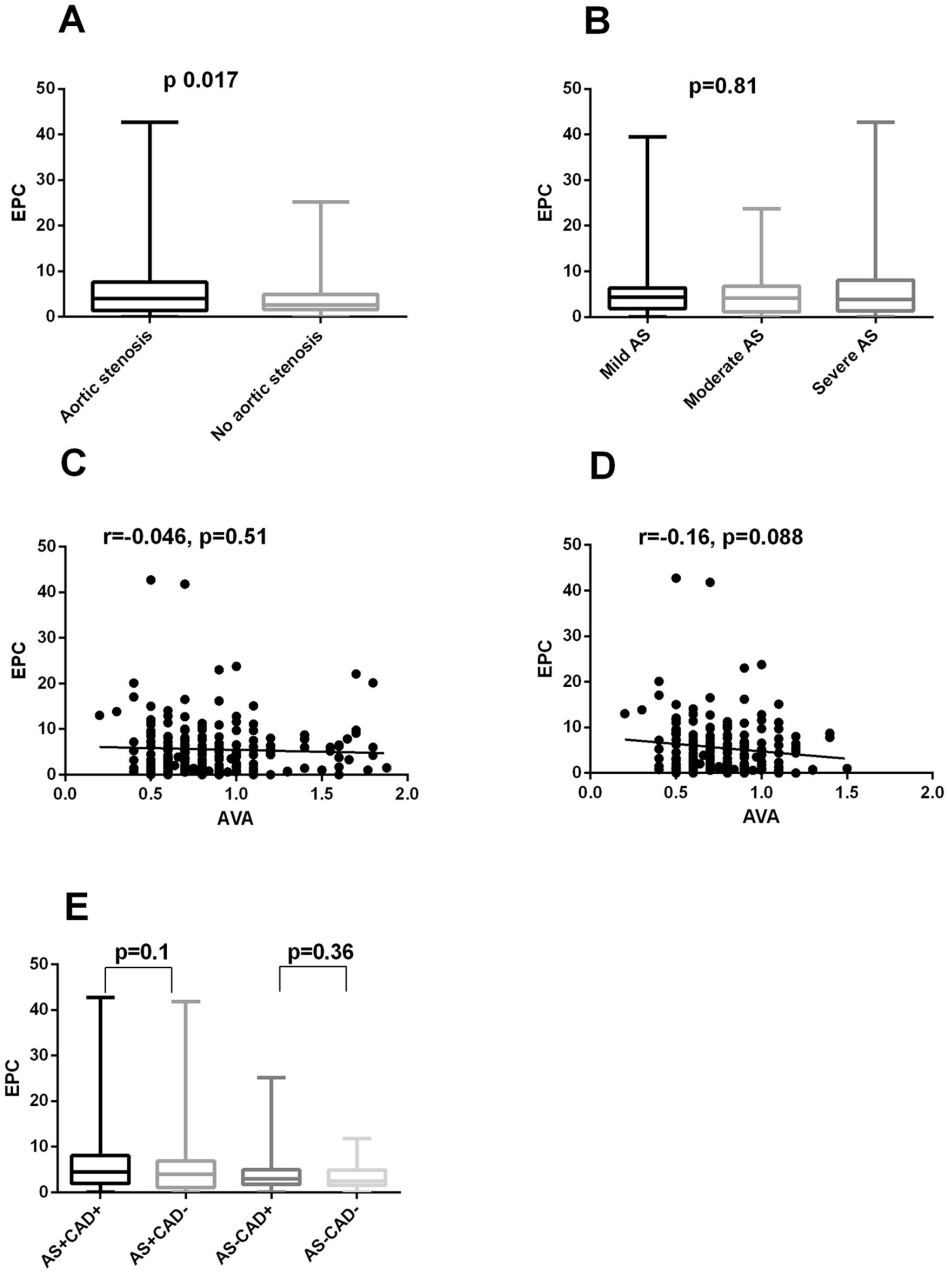
A. EPC levels in patients with AS and the control group. B. EPC levels in patients with mild, moderate and severe AS. C. Correlation between EPC level and aortic valve area in all AS patients. D. Correlation between EPC level and aortic valve area in moderate and severe AS patients. E. EPC level in patients with and without coronary artery disease in patients with AS and the control group.

Patients with AS had higher levels of EPCs as compared to the control group (4.08 (1.39–7.6) cells/μl vs 2.64 (1.59–4.94) cells/μl, p = 0.017, Fig 1a). On logistic regression analysis, after correcting for age, gender, medical therapy and hemoglobin level, only the Hg level and EPC levels correlated with AS (OR 0.67 95%CI 0.55–0.82, p<0.001 and OR 2.056, 95%CI 1.13–3.75, p = 0.019, respectively).

There was no difference in EPC level within the different AS etiologies (p = 0.28). There was no significant difference in EPC levels between patients with mild, moderate and severe AS (p = 0.81, Fig 1b) and no correlation was observed between EPC and aortic valve area (AVA) (r = -0.046, p = 0.51, Fig 1c). However, in the subgroup of patients with moderate and severe AS there was a borderline, not statistically significant, negative correlation between EPC level and aortic valve area (AVA) (r = -0.16, p = 0.09, Fig 1d).

EPC levels were also compared in patients with and without known CAD in patients with AS and in the control group. There was no significant difference in EPC number in patients with and without CAD in each group (p = 0.10 and p = 0.36, respectively, Fig 1e). We performed a subgroup analysis and included only patients with no evidence of CAD on coronary angiography (69 patients with AS and 32 patients in the control group). In this subgroup as well, there was a significant difference in EPC level, between patients with and without AS (3.8 (1–6.9) cells/μl vs 2.6 (1.5–3.4) cells/μl, p = 0.04). We also compared patients with severe AS without CAD and severe AS with severe CAD and did not find differences in EPC level in these subgroups of severe AS (5.39(1.9–8.04) vs 4.24(0.94–7.94), p = 0.54)

**Fig 2:**
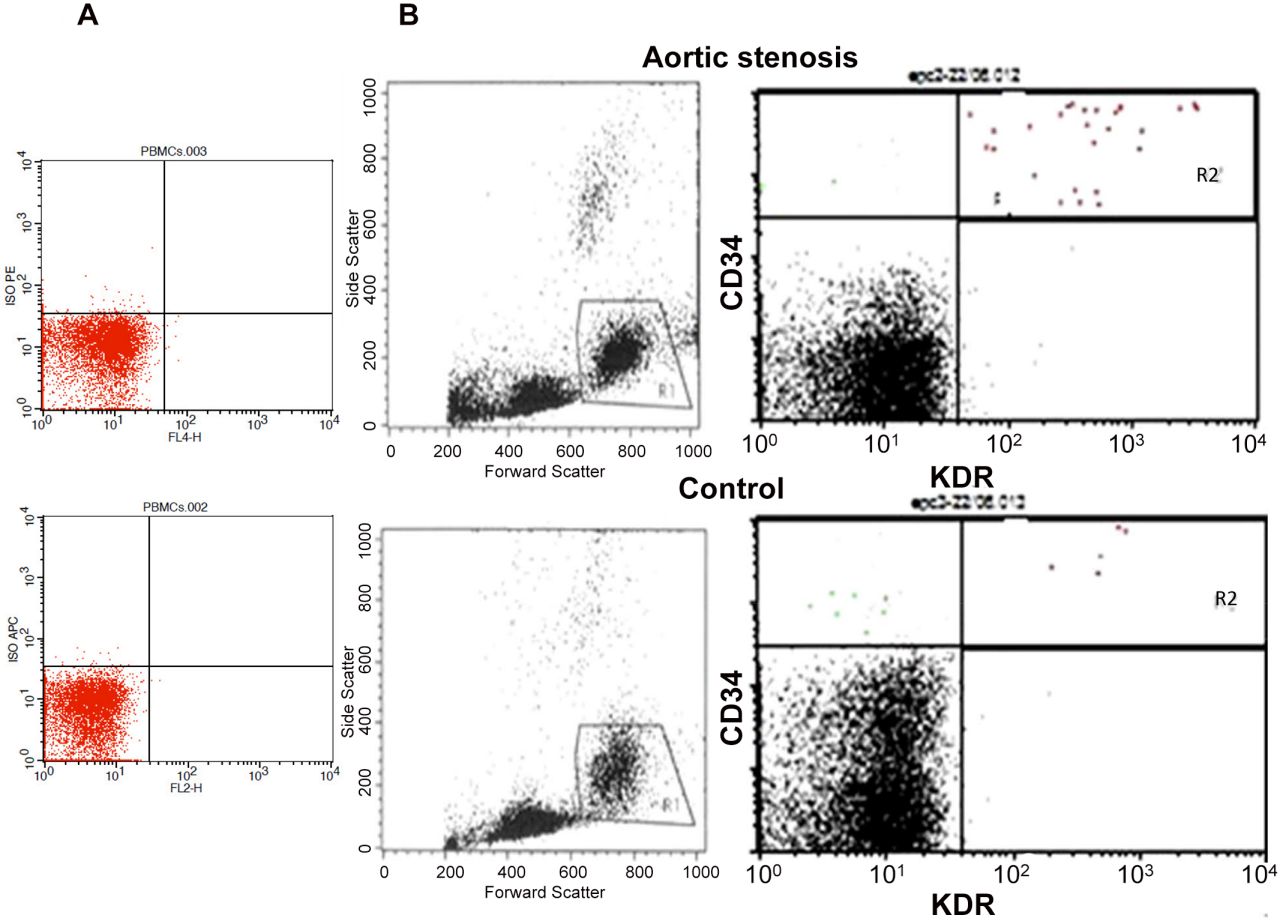
FACS analysis of circulating EPCs in patients with aortic valve stenosis (AS) and controls. **A.** Isotope control; B. A representative example of a FACS analysis for the quantification of CD34+/KDR+ cells in patient with AS and patient with no AS is shown. R1, viable cells; R2, cells positive for CD34 and KDR.

A representative flow cytometric evaluation of CD34+KDR+ cells in patients with and without AS is shown in Fig 2.

**Fig 3:**
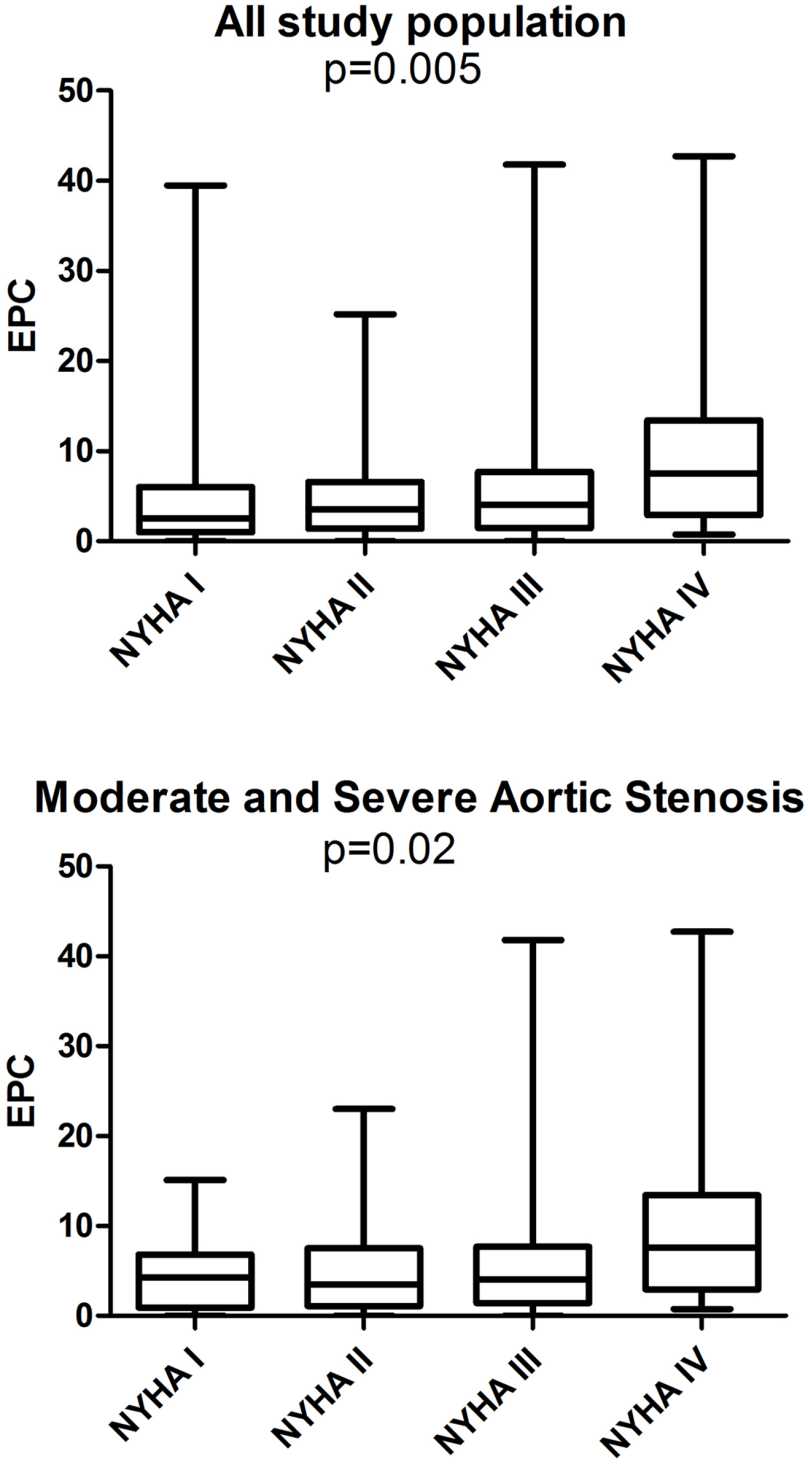
EPC level according to HYHA class in all study population and in patients with moderate and severe AS.

EPCs levels were higher in patients in NYHA class IV compared to NYHA class I. The difference in EPCs level in various NYHA classes was seen in all patients, as well as in patients with significant AS only (p = 0.005 and p = 0.02, respectively, Fig 3).

For outcome analysis the patients were categorized into two groups according to the median endothelial progenitor-cell count at the time of enrollment. One group with a high number of EPCs (4–43 cells/μl, median 7 cells/μl) and the second group with a low number of EPCs (0–3 cells/μl, median 1 cell/μl). The presence of AS and treatment with beta blockers were associated with a high baseline level of EPCs (p = 0.03 and p = 0.01, respectively).

### Clinical outcome in patients with AS

**Fig 4:**
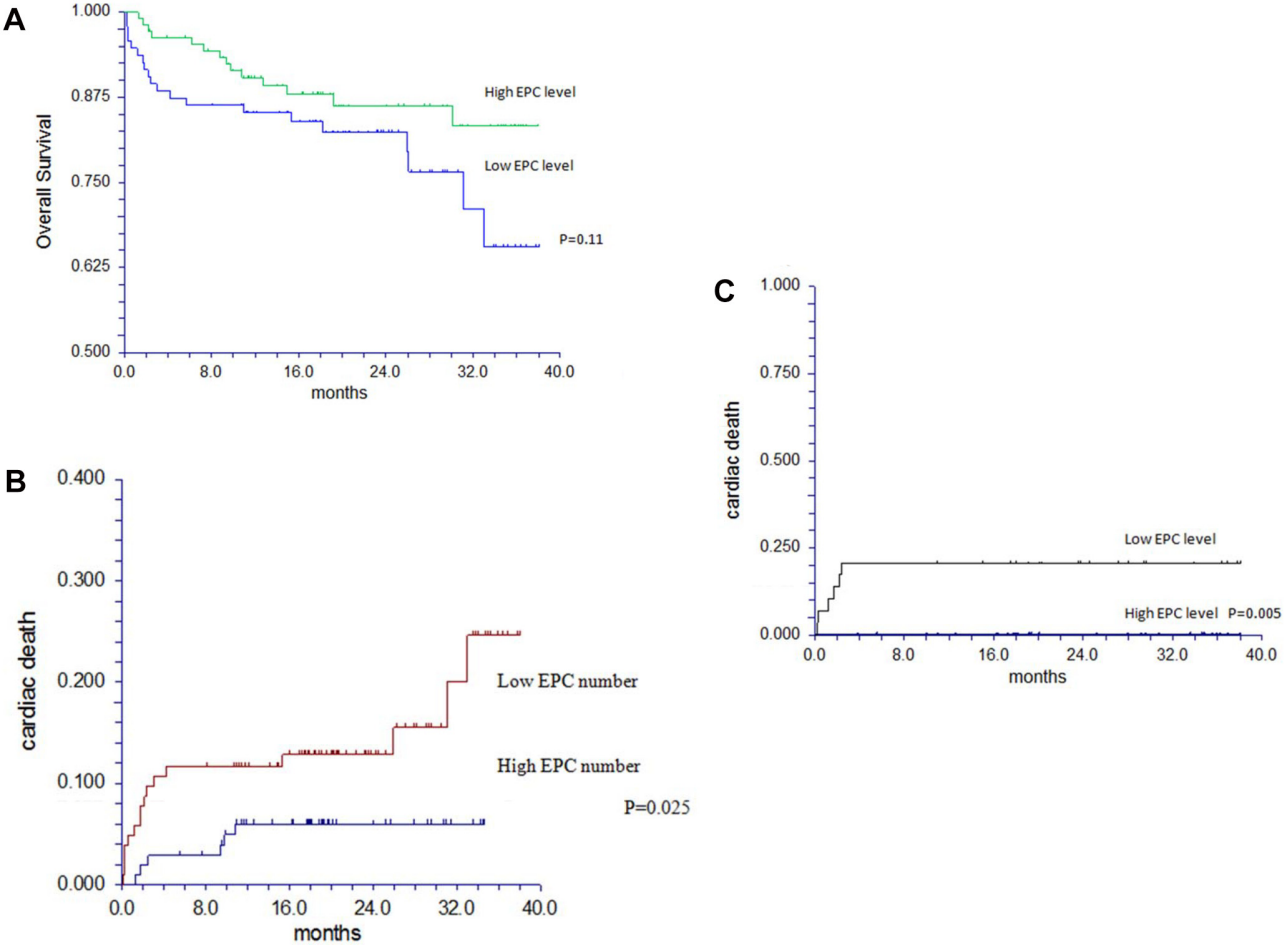
A. Kaplan Meier survival curve for all cause mortality in patients with moderate and severe AS in relation to EPCs. B. Cumulative incidence of cardiac death by EPC number in patients with AS. C. Cumulative incidence of cardiac death by EPC number in patients with AS and no CAD.

The mean follow up was 20 month (range 2.3–38). As expected, there was no need for valvular intervention and there were no deaths in patients with mild AS during follow up. In the group of 203 patients with moderate and severe AS, 55 patients (27%) underwent surgical aortic valve replacement (sAVR) and 65 patients (32%) underwent transcatheter aortic valve implantation (TAVI). Seventeen patients had no intervention, despite having a clinical indication, because of patient refusal or high risk due to concomitant illness. Thirty four patients (17%) with moderate or severe AS died during follow up, 17 of them (50%) had a prior intervention (8 sAVR and 9 TAVI). Twenty patients died of cardiac causes. Thirteen patients died from worsening CHF, 2 patients had fatal myocardial infarction and 5 patients died suddenly. Fourteen patients died due to non cardiac causes. Five patients died of cerebrovascular accident, 4 patients due to malignancy, 4 patients due to infections, and one of severe pulmonary disease. The overall 2 year survival was 85% (95%CI, 79–90). The level of EPCs was not related to the performance of intervention or to overall survival (p = 0.37 and p = 0.11 respectively, Table 2, Fig 4a). However, low EPC levels were associated with cardiac death (p = 0.03). The presence of coronary artery disease (CAD), functional class, AVA, pulmonary artery systolic pressure and EPC level were related to cardiac death in patients with moderate and severe AS, in the univariate analysis (Table 3). Since AVA, NYHA class and pulmonary artery systolic pressure are related clinically and statistically (r = -0.427, p<0.001, r = -0.28, p<0.001 and r = 0.402, p<0.001, respectively)) we performed factor analysis to calculate an index that included the 3 variables, the AS severity index. In a multivariate analysis model EPC level and AS severity index were significant predictors of cardiac death (HR 3.07, 95%CI 1.15–8.24, p = 0.026 and HR 1.79, 95%CI 1.04–3.08, p = 0.037, respectively). The cumulative incidence of cardiac death in relation to EPC level is shown in Fig 4b.

**Table 2:** Events during follow up.

	All	Group 1	Group 2	
AVR/TAVI	120	64	56	0.37
CARDIAC DEATH	20	6	14	0.03
ALL DEATH	34	17	20	0.11

AVR-Aortic valve replacement, TAVI–transcutaneous aortic valve implantation

**Table 3:** Cox regression model: variables as predictors of cardiovascular mortality.

UNIVARIAT ANALYSIS					MULTIVARIAT ANALYSIS			
	p	HR	95.0% CI for HR		P	HR	95.0% CI for HR	
			Lower	Upper			Lower	Upper
Age	.0510	1.057	1.000	1.118	.586	1.017	.956	1.082
Gender	.310	.635	.264	1.526				
Hypertension	.644	.787	.285	2.172				
Diabetes mellitus	.949	.971	.387	2.433				
Hyperlipidemia	.087	.463	.192	1.118				
Smoking	.280	.330	.044	2.464				
Coronary artery disease	.039	2.744	1.054	7.140	.115	2.179	.826	5.747
EPC	.034	2.818	1.082	7.338	.026	3.074	1.146	8.243
Hemoglobin (g/dL)	.225	.845	.643	1.109				
White blood cells (K/ul)	.094	.814	.640	1.035				
Platelets (K/ul)	.091	.993	.986	1.001				
Protein (g/dL)	.580	.822	.410	1.647				
Albumin (g/dL)	.785	.864	.303	2.464				
Ca+(mg/dL)	.177	.619	.309	1.242				
LDL (mg/dL)	.482	1.005	.990	1.020				
LVEDD (mm)	.716	.985	.909	1.068				
LVESD (mm)	.913	.994	.895	1.104				
LV mass (g)	.264	1.004	.997	1.011				
LVEF (%)	.913	.994	.895	1.104				
Peak AV gradient (mmHG)	.055	1.013	1.000	1.027				
Mean AV gradient (mmHG)	.085	1.019	.997	1.041				
AS severity index	0.005	1.831	1.196	2.803	0.037	1.785	1.035	3.077
AVA (cm^2^)	.005	.038	.004	.372				
PAP (mmHG)	.043	1.029	1.001	1.058				
NYHA class	.022	3.058	1.175	7.961				

LDL low density cholesterol, LVEDD-left ventricular end diastolic diameter, LVESD-Left ventricular end systolic diameter, LVEF-left ventricular ejection fraction, AV-aortic valve, AVA aortic valve area, PAP pulmonary artery press

Sixty four patients with moderate and severe AS had no CAD on coronary angiography. Six patients died due to cardiac causes during follow up; all had low number of EPCs. On univariate analysis, EPC level and LV mass were related to cardiac death in this subgroup (p = 0.043 and p = 0.026, respectively). The cumulative incidence of cardiac death in relation to EPC level in patients with AS and no CAD is shown in Fig 4c.

### EPC change after intervention

**Fig 5:**
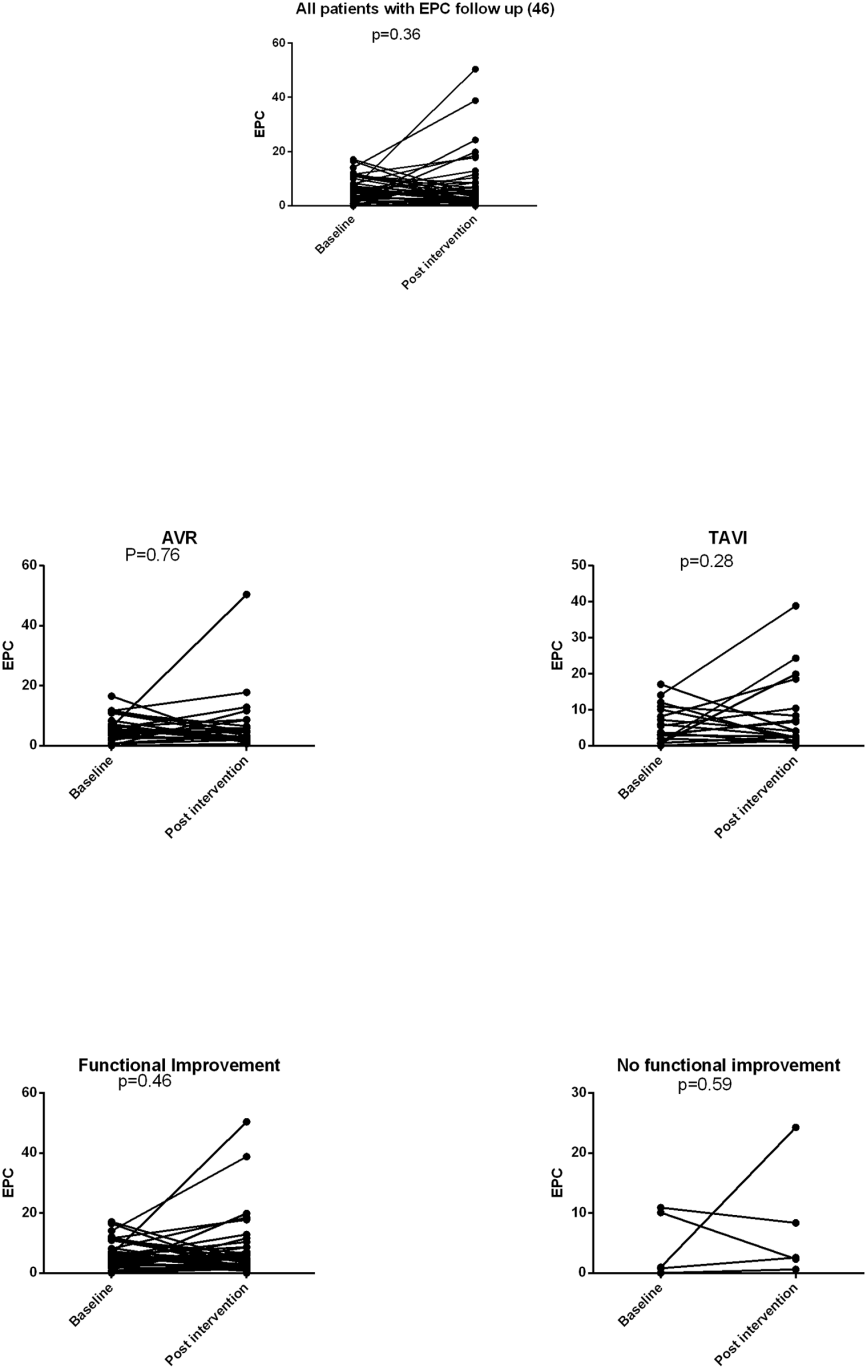
EPC level before and after intervention. EPC level before and after TAVI. EPC level before and after AVR. EPC level in patients with and without functional improvement. EPC level before and after intervtion in AS patinets without and with CAD.

EPC levels were measured during follow up in 46 patients, 3–12 (median 9) months after intervention. The mean difference in EPCs between baseline and after intervention was 1.5±10 cells/μl (p = 0.36, Fig 5). This observation was similar in the subgroups of patients who underwent surgical or transcutaneous aortic valve replacement (p = 0.76 and 0.28) respectively). There was no significant difference between patients that had an improved functional class and those who did not improve after intervention (p = 0.46 and p = 0.59), respectively), however the number of patients in each group was small. There was also no difference in EPC level before and after aortic valve intervention in patients without and with CAD assessed by coronary angiography (p = 0.21 and p = 0.78, respectively).

## Discussion

In the present study we have shown in a relatively large population of patients with AS, that in subjects with aortic stenosis there is an increased number of EPCs as compared to controls, irrespective to the presence of CAD. There was no significant change in EPC levels after aortic valve intervention. The present study is the first to show that levels of circulating EPC are an independent predictor of cardiac mortality in patients with aortic stenosis. During a mean observation of 20 months, a significantly lower incidence of cardiovascular death was observed in patients with high EPC numbers, irrespective of other known risk factors for cardiovascular death.

There is limited data on the role of EPC in aortic valve stenosis. Matsumoto et al studied whether degenerative AV stenosis is associated with the presence of valvular endothelial senescence and a reduction in the number and function of endothelial progenitor cells.[16] They reported that senescent ECs are present on the aortic side of degenerative AVs. They also compared circulating EPC numbers and function in peripheral blood samples from 15 patients with severe AS (CAD was excluded) with 18 age-matched controls and found that the number and the migratory capacity of EPCs are reduced. They suggested that the reduced levels of circulating EPCs may be the result of increased cell senescence as well as an enhanced apoptosis of EPCs in AS patients. These results suggest that in patients with AS, valvular endothelial cell regeneration may be impaired not only by an increased senescence of valvular endothelial cells but also by a reduced number and function of circulating EPCs. In contrast to this study, we have found elevated circulating EPC levels in patients with degenerative AS. There are significant differences between the two studies with respect to the design and patient characteristics. The number of patients in our study is significantly higher. Patients with CAD were allowed and the mean patient's age, the percent of patients with hyperlipidemia and the number of subjects on statin therapy were significantly higher. In addition, the former study assessed EPCs only in end stage aortic stenosis.

Gössl et al assessed the role of circulating EPCs with osteoblastic phenotype in human aortic valve calcification.[17] The study population included patients with mild and moderate AS without CAD and two groups of severe AS with and without CAD. They found low number of EPCs in patients with mild and moderate AS as well as in patients with severe AS and CAD. However in patients with severe AS with no CAD the EPC level was similar to the control group. The results of our study are different mainly in the subgroup of patients with mild and moderate AS and in the patients with severe AS and severe CAD. This may be explained by the different design of the studies, enrolling consecutive, real life patients with AS that in 40–50% have concomitant CAD and very frequently, associated endothelial dysfunction. The mean age of the patients in our control group was higher with more patients with diabetes. Our data showed no significant difference in EPCs within various AS severities irrespective of the presence of CAD.

Our results are in line with the study by Redondo et al that reported elevated CD34+kdr+ cell level in patients with severe AS before AVR compared to a control group.[18]

We found higher EPC level in patients with advanced functional class. This was not assessed in previous reports in patients with AS. Vlgimigli et al assessed EPCs in patients with various degrees of congestive heart failure.[26] All patients included had systolic dysfunction with low LVEF and the majority of patients had ischemic or idiopathic dilated cardiomyopathy. They found a biphasic correlation between EPC and functional class. The suggested explanation for these findings was increased bone marrow response to diffuse and severe endothelial damage in the early stage of the disease and a suppressive effect on hematopoesis, possibly by TNF-α, that counteracts and overwhelms the triggers for EPC release in advanced disease. We have previously assessed EPC numbers in patients with systolic and diastolic CHF and found a positive correlation between EPC and functional class.[27] The majority of the patients in our study had preserved systolic function and the etiology of CHF was mainly pressure overload due to AS and left ventricular hypertrophy with diastolic dysfunction. TNF- α was shown to be elevated in patients with pressure overload and also related to aortic valve calcification and progression.[28,29] However, the balance between the endothelial damage trigger and the hematopoietic suppression in patients with severe AS and various functional classes is not known.

There are various clinical and echocardiographic parameters that are known to be related to prognosis of patients with severe AS, symptomatic and asymptomatic.[19,30] These factors include symptoms, valve calcification, aortic valve peak velocity, LV hypertrophy and mass and LVEF. However, there are no previous reports on the prognostic role of EPC levels in patients with significant AS. Werner et al showed that in patients with CAD the level of circulating CD34+KDR+ EPCs predicts the occurrence of cardiovascular events and death and may help to identify patients at increased cardiovascular risk. [4] EPCs have also shown to play a significant role during myocardial repair following AMI and predicting myocardial viability.[31] In patients with heart failure, EPC levels were found to be an independent predictor of all cause mortality. [27]

The present study is the first to show that the levels of circulating EPCs are independent predictor of cardiovascular mortality in patients with significant AS. The destruction of the endothelial cell layers on the aortic valve may be one of the triggers to the progression of aortic valve stenosis. Normally, the endothelial cell layer is kept intact by the division of mature endothelial cells or by restoration with circulation EPCs. Skowasch et al assessed aortic valves from patients with degenerative AS and suggested that the presence of EPCs appears to be a novel biological hallmark in end-stage calcified AS.[15]

In valvular endothelial damage, inflammation, neovascularization and possibly calcifications together with pressure overload are all related to elevated EPC levels and extent of peripheral mobilization. In line with this pathophysiologic determinants we observed elevated EPC levels in patients with AS. However, it was the patients with low EPCs who had the poor cardiovascular prognosis. These phenomena may be explained by a failed or exhausted EPC response to the various stimuli that can be primary or secondary. It is possible that the EPCs in patients with poor prognosis have a primary defect and do not respond to the stimuli of endothelial damage, ischemia add pressure overload, or that the EPC response is blunted by factors such as TNF-∞ that reduces the number of EPCs in these patients.

There was no change in EPC levels after intervention, this was seen in patients that improved and did not improve after intervention. The lack of change in EPC level after intervention may support the possibility of primary EPC defect.

There are several limitations to this study. There is still ambiguity with regard to the appropriate method by which to characterize circulating EPCs. We have chosen looking at C34+/KDR+ cells as this population is the most highly studied in this context and the one associated more closely with circulating growth factor levels as previously described by us. [23] The number of total events in the FACS analysis was relatively low; however we excluded dead cells and used high quality antibodies. We assessed only the number of EPC and did not perform functional tests. It is possible that the EPCs are malfunctioning. The study was done on a large population and although important, it was complicated to perform functional studies that are also sometimes not very reproducible. Based on our experience and of others that have also abandoned these analyses, the tested population (day 5 or day 21 epc) is extremely heterogeneous and does not represent the subpopulation of CD34KDR that are the true EPC. This causes high variability of the results of functional tests and makes such comparisons invalid. Due to the small number of true CD34/KDR it is impossible to obtain appreciable number of this 'optimal' population for functional tests. The study population is heterogenic, with patients with risk factors and coronary disease in almost 50%. However these are real world, consecutive patients from our valvular clinic and the conclusions are related to the whole population. We also performed subgroup analysis for patients with no CAD. We did not measure EPC longitudinally during follow up, to assess whether the EPC levels in the same patients change with AS severity and symptoms. However, there is relatively high number of patients in each subgroup and it is possible to interpolate these changes in the EPCs. Finally, the event rate is relatively low, this is due to the fact that the patients were followed in valvular clinic and treated according to guidelines. In order to reduce the number of variables per event we used factor analysis to calculate an aortic stenosis severity index and used it in Cox regression analysis.

### Conclusions

Levels of circulating EPCs are increased in patients with mild, moderate and severe AS. However, in patients with moderate or severe AS a relatively low number of EPCs is associated with cardiac death at follow up. We show for the first time that circulating endothelial progenitor cells in patients with aortic stenosis can be used to identify patients at high risk for major adverse cardiac events.

## Abbreviations

AS: aortic stenosis
AV: aortic valve
AVR: aortic valve replacement
AVA: aortic valve area
CAD: coronary artery disease
CHF: congestive heart failure
EPC: endothelial progenitor cell
LVEF: left ventricular ejection fraction
NYHA: New York heart association
sAVR: surgical AVR
TAVI: trans-catheter aortic valve implantation
TNF-α: Tissue necrosis factor α

## References

[1] Iung B, Baron G, Butchart EG, Delahaye F, Gohlke-Barwolf C, et al (2003) A prospective survey of patients with valvular heart disease in Europe: The Euro Heart Survey on Valvular Heart Disease. Eur Heart J 24: 1231–1243. 12831818 10.1016/s0195-668x(03)00201-x

[2] Vasa M, Fichtlscherer S, Aicher A, Adler K, Urbich C, et al (2001) Number and migratory activity of circulating endothelial progenitor cells inversely correlate with risk factors for coronary artery disease. Circ Res 89: E1–7. 11440984 10.1161/hh1301.093953

[3] Hill JM, Zalos G, Halcox JP, Schenke WH, Waclawiw MA, et al (2003) Circulating endothelial progenitor cells, vascular function, and cardiovascular risk. N Engl J Med 348: 593–600. 12584367 10.1056/NEJMoa022287

[4] Werner N, Kosiol S, Schiegl T, Ahlers P, Walenta K, et al (2005) Circulating endothelial progenitor cells and cardiovascular outcomes. N Engl J Med 353: 999–1007. 16148285 10.1056/NEJMoa043814

[5] Stewart BF, Siscovick D, Lind BK, Gardin JM, Gottdiener JS, et al (1997) Clinical factors associated with calcific aortic valve disease. Cardiovascular Health Study. J Am Coll Cardiol 29: 630–634. 9060903 10.1016/s0735-1097(96)00563-3

[6] Otto CM, Kuusisto J, Reichenbach DD, Gown AM, O'Brien KD (1994) Characterization of the early lesion of 'degenerative' valvular aortic stenosis. Histological and immunohistochemical studies. Circulation 90: 844–853. 7519131 10.1161/01.cir.90.2.844

[7] O'Brien KD, Reichenbach DD, Marcovina SM, Kuusisto J, Alpers CE, et al (1996) Apolipoproteins B, (a), and E accumulate in the morphologically early lesion of 'degenerative' valvular aortic stenosis. Arterioscler Thromb Vasc Biol 16: 523–532. 8624774 10.1161/01.atv.16.4.523

[8] Davies PF, Passerini AG, Simmons CA (2004) Aortic valve: turning over a new leaf(let) in endothelial phenotypic heterogeneity. Arterioscler Thromb Vasc Biol 24: 1331–1333. 15297285 10.1161/01.ATV.0000130659.89433.c1

[9] Dimmeler S, Zeiher AM (2004) Vascular repair by circulating endothelial progenitor cells: the missing link in atherosclerosis? J Mol Med (Berl) 82: 671–677. 15322703 10.1007/s00109-004-0580-x

[10] Deanfield JE, Halcox JP, Rabelink TJ (2007) Endothelial function and dysfunction: testing and clinical relevance. Circulation 115: 1285–1295. 17353456 10.1161/CIRCULATIONAHA.106.652859

[11] Sorrentino SA, Bahlmann FH, Besler C, Muller M, Schulz S, et al (2007) Oxidant stress impairs in vivo reendothelialization capacity of endothelial progenitor cells from patients with type 2 diabetes mellitus: restoration by the peroxisome proliferator-activated receptor-gamma agonist rosiglitazone. Circulation 116: 163–173. 17592079 10.1161/CIRCULATIONAHA.106.684381

[12] Mazzone A, Epistolato MC, De Caterina R, Storti S, Vittorini S, et al (2004) Neoangiogenesis, T-lymphocyte infiltration, and heat shock protein-60 are biological hallmarks of an immunomediated inflammatory process in end-stage calcified aortic valve stenosis. J Am Coll Cardiol 43: 1670–1676. 15120829 10.1016/j.jacc.2003.12.041

[13] Freeman RV, Otto CM (2005) Spectrum of calcific aortic valve disease: pathogenesis, disease progression, and treatment strategies. Circulation 111: 3316–3326. 15967862 10.1161/CIRCULATIONAHA.104.486738

[14] Mirzaie M, Meyer T, Schwarz P, Lotfi S, Rastan A, et al (2002) Ultrastructural alterations in acquired aortic and mitral valve disease as revealed by scanning and transmission electron microscopical investigations. Ann Thorac Cardiovasc Surg 8: 24–30. 11916439

[15] Skowasch D, Schrempf S, Wernert N, Steinmetz M, Jabs A, et al (2005) Cells of primarily extra-valvular origin in degenerative aortic valves and bioprostheses. Eur Heart J 26: 2576–2580. 16115807 10.1093/eurheartj/ehi458

[16] Matsumoto Y, Adams V, Walther C, Kleinecke C, Brugger P, et al (2009) Reduced number and function of endothelial progenitor cells in patients with aortic valve stenosis: a novel concept for valvular endothelial cell repair. Eur Heart J 30: 346–355. 10.1093/eurheartj/ehn501 19010796

[17] Gossl M, Khosla S, Zhang X, Higano N, Jordan KL, et al (2012) Role of circulating osteogenic progenitor cells in calcific aortic stenosis. J Am Coll Cardiol 60: 1945–1953. 10.1016/j.jacc.2012.07.042 23062532 PMC4213791

[18] Redondo S, Gonzalez-Rocafort A, Navarro-Dorado J, Ramajo M, Hristov M, et al (2012) Decreased pre-surgical CD34+/CD144+ cell number in patients undergoing coronary artery bypass grafting compared to coronary artery disease-free valvular patients. J Cardiothorac Surg 7: 2 10.1186/1749-8090-7-2 22214418 PMC3268732

[19] Nishimura RA, Otto CM, Bonow RO, Carabello BA, Erwin JP 3rd, et al (2014) 2014 AHA/ACC guideline for the management of patients with valvular heart disease: a report of the American College of Cardiology/American Heart Association Task Force on Practice Guidelines. J Am Coll Cardiol 63: e57–185. 10.1016/j.jacc.2014.02.536 24603191

[20] Lang RM, Bierig M, Devereux RB, Flachskampf FA, Foster E, et al (2005) Recommendations for chamber quantification: a report from the American Society of Echocardiography's Guidelines and Standards Committee and the Chamber Quantification Writing Group, developed in conjunction with the European Association of Echocardiography, a branch of the European Society of Cardiology. J Am Soc Echocardiogr 18: 1440–1463. 16376782 10.1016/j.echo.2005.10.005

[21] Quinones MA, Otto CM, Stoddard M, Waggoner A, Zoghbi WA, et al (2002) Recommendations for quantification of Doppler echocardiography: a report from the Doppler Quantification Task Force of the Nomenclature and Standards Committee of the American Society of Echocardiography. J Am Soc Echocardiogr 15: 167–184. 11836492 10.1067/mje.2002.120202

[22] Baumgartner H, Hung J, Bermejo J, Chambers JB, Evangelista A, et al (2009) Echocardiographic assessment of valve stenosis: EAE/ASE recommendations for clinical practice. Eur J Echocardiogr 10: 1–25. 10.1093/ejechocard/jen303 19065003

[23] George J, Shmilovich H, Deutsch V, Miller H, Keren G, et al (2006) Comparative analysis of methods for assessment of circulating endothelial progenitor cells. Tissue Eng 12: 331–335. 16548691 10.1089/ten.2006.12.331

[24] Massa M, Rosti V, Ferrario M, Campanelli R, Ramajoli I, et al (2005) Increased circulating hematopoietic and endothelial progenitor cells in the early phase of acute myocardial infarction. Blood 105: 199–206. 15345590 10.1182/blood-2004-05-1831

[25] Thomas HE, Redgrave R, Cunnington MS, Avery P, Keavney BD, et al (2008) Circulating endothelial progenitor cells exhibit diurnal variation. Arterioscler Thromb Vasc Biol 28: e21–22. 10.1161/ATVBAHA.107.160317 18296584

[26] Valgimigli M, Rigolin GM, Fucili A, Porta MD, Soukhomovskaia O, et al (2004) CD34+ and endothelial progenitor cells in patients with various degrees of congestive heart failure. Circulation 110: 1209–1212. 15249502 10.1161/01.CIR.0000136813.89036.21

[27] Michowitz Y, Goldstein E, Wexler D, Sheps D, Keren G, et al (2007) Circulating endothelial progenitor cells and clinical outcome in patients with congestive heart failure. Heart 93: 1046–1050. 17277352 10.1136/hrt.2006.102657 PMC1955007

[28] Kapadia SR, Yakoob K, Nader S, Thomas JD, Mann DL, et al (2000) Elevated circulating levels of serum tumor necrosis factor-alpha in patients with hemodynamically significant pressure and volume overload. J Am Coll Cardiol 36: 208–212. 10898436 10.1016/s0735-1097(00)00721-x

[29] Yu Z, Seya K, Daitoku K, Motomura S, Fukuda I, et al (2011) Tumor necrosis factor-alpha accelerates the calcification of human aortic valve interstitial cells obtained from patients with calcific aortic valve stenosis via the BMP2-Dlx5 pathway. J Pharmacol Exp Ther 337: 16–23. 10.1124/jpet.110.177915 21205918

[30] Bhattacharyya S, Hayward C, Pepper J, Senior R (2012) Risk stratification in asymptomatic severe aortic stenosis: a critical appraisal. Eur Heart J 33: 2377–2387. 10.1093/eurheartj/ehs190 22766580

[31] Lamirault G, Susen S, Forest V, Hemont C, Parini A, et al (2013) Difference in mobilization of progenitor cells after myocardial infarction in smoking versus non-smoking patients: insights from the BONAMI trial. Stem Cell Res Ther 4: 152 24423369 10.1186/scrt382 PMC4054959

